# A comparative study of blood pressure submission between mobilec app users and non-users in Romania: a pilot cohort study

**DOI:** 10.3389/fresc.2025.1580991

**Published:** 2025-04-25

**Authors:** Liviu Ionut Serbanoiu, Stefan Sebastian Busnatu, Andreea Elena Lacraru, Maria Alexandra Pana, Suzana Guberna, Dragos Trache, Plesu Elena, Catalina Liliana Andrei, Crina Sinescu

**Affiliations:** ^1^ Department of Cardiology, “Carol Davila” University of Medicine and Pharmacy, Bucharest, Romania; ^2^ Department of Cardiology, Emergency Hospital “Bagdasar-Arseni”, Bucharest, Romania

**Keywords:** telemonitoring, prevention, mobile application, cardiovascular diseases, blood preasure

## Abstract

**Introduction:**

Cardiovascular diseases (CVD) represent a major public health concern in Romania. Despite the importance of home blood pressure (BP) monitoring, adherence to this practice remains limited. With the growing availability of eHealth solutions, this study aimed to evaluate the user experience and engagement of individuals with and without CVD in using telemonitoring technology.

**Methods:**

A prospective cohort study was conducted involving 24 participants who used a smartwatch application for telemonitoring. In addition, 176 participants who did not use the application were surveyed through an online questionnaire to serve as a comparison group. Participants were included regardless of CVD status. Data collected included blood pressure submission frequency, app usage metrics, and daily step counts. The cohort using the smartwatch app was observed over a 60-day period.

**Results:**

Among the participants, 58.3% were male and 41.7% were female, with a mean age of 50.57 years. The average number of active app usage days was 47.5 out of 60, and blood pressure was submitted on average 31.79 days. The average daily step count was 4,624 steps. In comparison, the reference group (non-app users) submitted BP data on average 7.41 days out of 60. A positive correlation was observed between active usage and BP submissions in the app group, indicating higher engagement with the telemonitoring intervention. Variability in user involvement was noted, with moderate but periodic participation.

**Discussion:**

This pilot study suggests that Romanian patients are more likely to engage with mobile health technologies for hypertension management compared to traditional care approaches. The use of telemonitoring devices was associated with greater adherence to vital sign reporting. However, limitations such as small sample size, potential self-selection bias, and lack of randomization must be considered. Further research with larger, randomized control trials and extended follow-up is necessary to validate these findings. Empowering patients through eHealth solutions, alongside clinician support, may help mitigate the burden of cardiovascular diseases in Romania.

## 1 Introduction

Heart and blood vessel problems, collectively referred to as cardiovascular diseases (CVDs), include rheumatic heart disease, coronary heart disease, cerebrovascular illness, and other ailments. Following stroke (17.3%), other CVD (16.8%), and coronary heart disease (CHD) (41.2%), these conditions accounted for the majority of CVD-related fatalities in the US in 2020 (1–3). Cardiovascular diseases (CVD) constitute a serious global public health concern, and Romania is no exception. With the rising burden of heart-related illnesses, there is a growing demand for novel techniques for monitoring and treating cardiac health. CVD presents a major global fiscal burden on the healthcare system. In Romania, CVDs are a primary cause of mortality, contributing to approximately 60% of annual fatalities (4). Hypertension affects approximately 30% of Romanian adults (5) and leads to an increased risk of stroke, heart attack, heart failure, and other complications when not well controlled. The rise in heart-related illnesses has increased the demand for novel techniques to effectively monitor and manage cardiac health.

Hypertension, a major risk factor for cardiovascular disease, affects nearly 40.41% (6) of Romanian adults according to a study by Roşu and Mota (1, 7). When left uncontrolled, hypertension significantly increases the risk of stroke, myocardial infarction, heart failure, and other severe complications. Thus, improved management strategies for patients with hypertension and cardiac diseases have become a major public health priority in Romania. Traditionally, patients have had to visit healthcare facilities regularly for these assessments, which can be inconvenient and may lead to monitoring gaps (8).

To manage and avoid issues related to CVDs, it is essential to continuously monitor important measures such as blood pressure, heart rate, and electrocardiograms (ECGs) (2). Patients often have to make frequent trips to medical institutions for these evaluations, which may be difficult and result in treatment gaps, particularly for those who live in rural or disadvantaged regions (3). As a result, telemonitoring has become a viable method for improving cardiac patients' treatment from a distance thanks to advancements in digital health technology. Despite the growing global interest in eHealth solutions for chronic disease management, there is limited research on the use of telemonitoring apps in Eastern European populations, particularly Romania. Most telemonitoring studies have been conducted in Western Europe and the United States (9).

Telehealth adoption and effectiveness rely on technological infrastructure, resources, provider workflow integration, and patient preparedness (10). The practicality and usefulness of cardiac telemonitoring in Romania is debatable. Qualitative research has shown Romanian patients' preferences for in-person therapy, privacy issues, and technological limitations (11). Our study is the first in Romania to assess the use of a mobile health solution, specifically for blood pressure monitoring in patients with and without CVD. Do individuals with cardiovascular disease (CVD) utilize telemonitoring apps and input their blood pressure more often over two months than those without CVD, who were recruited via an online questionnaire? We hypothesized that having a health monitoring app with a cardiac diagnostic would boost telemonitoring compliance and adherence owing to the app's availability on mobile phones or digital watches. The viability of cardiac telemonitoring as an effective cardiovascular health management tool for Romanian patients will be assessed, considering compliance and potential benefits.

To assess this, we recruited adult Romanian patients from cardiology clinics, with and without a diagnosis of CVD. The solution included the provision of a watch app. This study takes a fresh approach by evaluating compliance with blood pressure submission not only in individuals with CVD but also in those without a CVD diagnosis, which is an underserved group in telemonitoring research. By comparing compliance in these two groups, this research could demonstrate the wider relevance of telemonitoring technologies, not only for controlling known illnesses, but also for avoiding complications in at-risk individuals. Furthermore, this study adds to the increasing body of data supporting telemonitoring as a cost-effective and scalable technique with the potential to lower the total healthcare burden. This comparison allowed us to determine if telemonitoring solutions motivate both groups, that is, individuals with or without CVD, to follow their monitoring schedules equally well, which would be beneficial in understanding what measures could be implemented to adhere the patients to regular cardiac health monitoring in future studies. The secondary outcomes included average heart rate, average daily steps, and SaO2 levels taken to track physical activity in both CVD and non-CVD patients.

The novelty of this studies lies in its focus on Eastern European population particularly Romania where telemonitoring adoption remains underexplored compared to other regions of the world This study explores telemonitoring adoption in Romania, focusing on blood pressure monitoring using a mobile application. It compares engagement and compliance of patients with and without CVD, shedding light on telemonitoring's applicability for disease management and possible preventive care. These findings can guide the development of tailored telehealth strategies and policies to better serve the needs of Romanian cardiac patients.

## 2 Materials and methods

### 2.1 Study design and objectives

This study aimed to evaluate in-person cardiac rehabilitation and telemedicine services in Romania. This prospective observational study was conducted in collaboration with Bagdasar Arseni Emergency Hospital and a telemonitoring mobile app. The medical team responsible for conducting the research and processing data was affiliated with the “Carol Davila” University of Medicine and Pharmacy in Bucharest, Romania.

The mobile app was developed by the authors in collaboration with two engineers. This is not an existing app. In this study the mobile app was used exclusively for remote monitoring of patients' different parameters, without providing personalized medical recommendations. Users received regular notifications reminding them to measure their blood pressure and manually enter the values into the app. The recorded data was stored in a digital journal, allowing patients to track their progress over time.

The app did not offer automatic data interpretation or medical alerts based on the entered values. Additionally, it did not generate recommendations for treatment adjustments or lifestyle modifications. Its primary role was to facilitate self-monitoring of vital parameters*, providing patients with a simple tool to organize and review their own measurements. Hence, the purpose was to assess people with CVD who underwent blood pressure readings for a duration of 2 months as compared to those without the telemonitoring app on their watches. The data (SaO2, HR, and steps) were collected automatically using the mobile app; however, for blood pressure, a push-notification system was used to remind the patients to enter the blood pressure manually.

The following inputs and G*Power software were used to perform independent *t*-tests:

*α* = 0.05, power = 0.80, and *d* = 1.41. A total of 52 samples were needed. The research was not sufficiently powered because the actual sample size in the mobile app group was 24, and the questionnaire group had 176 participants without telemonitoring. The inclusion criteria for the 24 patients included in the telemonitoring program were as follows: diagnosed with NYHA Class I–III heart failure; 13 out of 24 patients had heart failure. Exclusion criteria via the mobile app was unstable angina; resting systolic blood pressure >200 mmHg, diastolic blood pressure >110 mmHg, a drop in blood pressure of more than 20 mmHg upon standing, severe aortic stenosis, septicemia, uncontrolled arrhythmias, uncontrolled atrial tachycardia with a heart rate >120 bpm, decompensated heart failure, third-degree atrioventricular block, recent pulmonary thromboembolism, phlebitis, persistent ST segment elevation greater than 2 mm, uncontrolled diabetes mellitus, locomotor disabilities, thyroiditis, hypokalemia, hyperkalemia, hypervolemia, inability to understand and adhere to the protocol, and/or to give informed consent. Regarding the 176 individuals who responded to the online questionnaire, the inclusion criteria were: Willingness to participate in the study. The exclusion criteria were as follows: inability to understand and adhere to the protocol and/or to give informed consent, refusal to consent to the processing of personal data samples' *post hoc* power >0.99. The sample of 24 app patients did not have a sufficient power of over 0.80 at an alpha of 0.05, or a substantial effect size (Cohen's *d* = 1.41) for the difference in BP submission frequency across groups. As a result, the research did not have sufficient statistical power to identify significant variations in the main results.

## 3 Data collection

### 3.1 Structure of the survey questionnaire

The “Carol Davila” University of Medicine and Pharmacy medical staff carefully created the questionnaire, which was distributed via the Google Forms application. It was published online, including on several social media sites, where it was viewable for ten weeks, especially from September 13 to September 14, 2023.

The survey's six items included questions on social, demographic, and medical data, as well as tests of respondents' knowledge of cardiovascular health.

Questionnaire Structure:

1. Do you agree to participate in the study?
2. I have read the personal data processing policy and agree to its processing.
3. What is your age range?
4. Please specify your gender.
5. Do you know yourself with any cardiovascular pathology?
6. How many times have you had your blood pressure checked in the last 60 days?

To ensure consistency in data collection, only two items were allowed, with the majority of replies to the questionnaire consisting of predetermined yes/no choices or multiple-choice alternatives.

### 3.2 Statistical analysis

Statistical analyses were performed using IBM SPSS Statistics version 26. Descriptive statistics, including means, medians, standard deviations, and ranges, were calculated for all continuous variables (app usage metrics, physiological parameters, and BP submissions). Independent samples *t*-tests were used to compare mean BP submission frequencies between the telemonitoring group (*n* = 24) and non-user group (*n* = 176). Pearson correlation analysis assessed the relationship between active app days and BP submissions. Frequency distributions and percentages were computed for categorical variables (CVD status, BP submission categories. Tests were two-tailed as well with statistical significance set at *p* < 0.05. Effect sizes were reported where applicable to complement *p*-value interpretation.

## 4 Results

We conducted the study on 24 patients who had a telemonitoring app and 176 patients who did not use a telemonitoring app and were recruited using an online questionnaire. In the telemonitoring group, 13 (54.2%) of the 24 individuals had previously been diagnosed with cardiovascular disease (CVD), whereas 11 (45.8%) had not. We included patients with and without CVD, as the sample size was already small. In terms of sex distribution, the research sample consisted of 10 females (41.7%) and 14 males (58.3%). This suggests that there were more men than women among participants. Additionally, given that more than half of the participants already had CVD, this shows that despite both groups being represented, cardiac patients made up a larger portion of the study population than healthy controls.

There were 24 patients in the sample, and comprehensive information was available for both average heart rate and oxygen saturation (SaO2). There were no missing data. Demographic and clinical characteristics of participants are summarized in Table 1. The average heart rate varied among the patients. The mean value was 80 beats/min. The particular mean and median heart rates cannot be determined from these data in terms of central tendency; however, based on the lowest, maximum, and mode values, they are most likely to range between 70 and 80 bpm.

**Table 1:** Demographic and clinical characteristics of participants.

Characteristic	Mobile app users (*N* = 24)	Non-app users (*N* = 176)
Age (mean ± SD)	46.04 ± 16.31 years	Not reported
Sex (% male)	58.3%	Not reported
CVD Diagnosis (%)	54.2% (NYHA I–III)	39.8%
Key Exclusion Criteria	Unstable angina, severe hypertension, etc.	N/A

The oxygen saturation percentages for SaO2 had a range of 6% points, going from 93% at the lowest to 99% at the highest. For SaO2, there were many modes, the smallest being 94%. This shows that, among these individuals, 94% oxygen saturation was the most common. Although the mean and median cannot be established with accuracy, they are most likely to be between 96 and 97 percent based on the middle of the range and several modes in the mid-90 s.

### 4.1 Demographic and clinical characteristics of participants

24 patients with valid heart rate readings were included in the study. The heart rates ranged from 62 to 99 beats per minute (bpm) according to the frequency distribution. The heart rate and SaO2 measurements are summarized in Table 2. There were no gaps in the distribution and each number from 62 to 99 appeared at least once.

**Table 2:** Descriptive statistics for heart rate and oxygen saturation.

Statistic	Heart rate (bpm)	SaO2 (%)
*N* Valid	24	24
Missing	0	0
Mode	80	94
Range	37	6
Minimum	62	93
Maximum	99	99

Note: For SaO2, multiple modes exist. The smallest value (94) is shown.

Three individuals (12.5%) had a heart rate of 80 bpm, which was the most frequent. The next two values, which were recorded for two patients (8.3% of the sample), were 81 and 92 bpm. The other 21 heart rate readings, between 62 and 87 and 89 and 99 beats per minute, occurred only once for one patient (4.2% of the sample).

The heart rate value dividing the lowest 25% of the distribution from the highest 75% (first quartile) was 78 bpm, expressed in percentiles. The median heart rate of 82 bpm was the lowest among the top 50% of the heart rates. The heart rate in the third quartile, which was 91 bpm, was the range below which 75% of the observations occurred.

This aggregated frequency data cannot be used to calculate the mean and standard deviation conclusively. However, the predicted mean is most likely to be approximately 80 bpm, with a standard deviation of approximately 10 bpm based on the lowest (62 bpm), maximum (99 bpm), quartiles, and frequency distribution.

The heart rate frequency table shows a generally wide range of values from 60 to 90 bpm. The distribution lacked a significant skew and was symmetric. The range and variability were enhanced by a couple of higher outliers in the 90 s and by one at 99 bpm. For this heart rate data, further statistical analysis may provide exact measurements of central tendency and variance. However, frequency distribution provides an in-depth analysis of the spread, shape, and most prevalent values in Table 3.

**Table 3:** Frequency distribution of heart rate (bpm).

Heart rate (bpm)	Frequency	Percent
62	1	4.2%
67	1	4.2%
73	1	4.2%
74	1	4.2%
76	1	4.2%
77	1	4.2%
78	1	4.2%
80	3	12.5%
81	2	8.3%
82	1	4.2%
84	1	4.2%
85	1	4.2%
87	1	4.2%
89	1	4.2%
90	1	4.2%
91	2	8.3%
92	2	8.3%
93	1	4.2%
99	1	4.2%
Total	24	100.0%

24 individuals made up this sample, and all three app engagement measures—active days, blood pressure submissions, and daily average steps—were fully documented. There were no values missing.

Participants used the app actively, on average, for 47.5 of the 60 days, with a median of 57.5 days. There was a broad range of activity, from just 7 days to a maximum of 60 days. Utilization days showed significant variation, with a standard deviation of 17.26. The mean number of days for blood pressure submissions was 31.79, which was somewhat longer than the median of 30 days. Values with a standard deviation of 18.93 varied from two submission days to 60 days. This suggests that the frequency of blood pressure tracking is heterogeneous. Descriptive statistics for app engagement metrics are reported in Table 4. This variation could be due to various factors that we intend to explore in future studies.

**Table 4:** Comparison of blood pressure submission frequency between telemonitoring app users and non-users.

Parameter	Mobile users (*N* = 24)	Non-users (*N* = 176)
Mean BP submissions	31.79	7.41
Median BP submissions	30	Unknown
Range (submissions)	2–60	0–120
Submission frequency	Daily to weekly	Rare (67.6% ≤4 submissions)

Every day, the participants took an average of 4,624 steps. The middle number of 4,523 steps is more normal, considering the effect of outliers. In a wide range, there were steps of just over 1,000 to more than 10,000. The standard deviation of 2,304 steps also shows that the variability can be affected by both very high and very low step counts. The wide standard deviations, low means, and wide ranges for all three measures showed that people's use of the mobile health app was very different. Based on the median and middle 50% of the data, most users were moderately busy and occasionally submitted their BPs. They also took between 3,000 and 9,000 steps/day. The findings are summarized in Table 5. However, extreme outliers with high involvement levels increased the average and variance. In contrast to regular daily contact over the course of two months, the data revealed variations in app usage habits. Further investigation is required to understand the variables that influence the engagement patterns.

**Table 5:** Descriptive statistics for app engagement metrics.

Statistic	Active days	BP submitted	Average steps/day
*N* Valid	24	24	24
Missing	0	0	0
Mean	47.50	31.79	4,624.52
Median	57.50	30.00	4,522.77
Std. deviation	17.26	18.94	2,304.35
Range	53	58	9,273.12
Minimum	7	2	1,011.86
Maximum	60	60	10,284.98

To determine the link between these two engagement indicators, Pearson correlation analysis was performed on the number of active days using the telemonitoring app and the frequency of blood pressure (BP) uploads. Active days and BP submissions had a statistically significant and relatively strong positive correlation (*r* = .564, *p* = .004). The Pearson correlation coefficients and significance levels are shown in Table 6.

**Table 6:** Correlations between active days and BP submissions.

	BP_Submitted	Active_Days	
BP_Submitted	Pearson Correlation	1	.564*
Sig. (2-tailed)		.004	
*N*	24	24	
Active_Days	Pearson Correlation	.564*	1
Sig. (2-tailed)	.004		
*N*	24	24	

* Correlation is significant at the 0.01 level (2-tailed).

This suggests a clear relationship between participants' active app use days and the regularity with which they recorded their blood pressure readings; when one grew, the other did too. According to the coefficient of determination (*r*^2^ = .5642 = .3182), active days contributed to approximately 32% of the variation in BP submissions.

Correlation analysis revealed a modest but significant link between participants' use of the telemonitoring app and their frequency of blood pressure reporting. More active days were associated with higher BP submissions, as indicated by the positive *r*-value. This supports our prediction that participants will concurrently exhibit greater app-measure engagement than less actively engaged users. Further regression analysis is necessary to forecast the BP submission frequency from active days.

### 4.2 Patients without telemonitoring system

After establishing the association between telemonitoring activities and BP submission, we wanted to know how often patients without the app submitted BP over two months. We questioned 176 individuals with or without CVD how often they checked their blood pressure over two months. 176 people were included in the questionnaire group. Whether the person had any CVD conditions is indicated by CVD. A “yes” answer indicated a CVD diagnosis, but a “no” response did not. Out of 176 eligible cases, 70 (39.8%) had CVD, whereas 106 (60.2%) did not. To be precise, 39.8% of the sample said yes. Based on the sample, little less than 4 in 10 had CVD. The sample included 60.2% NO responses. This shows that nearly three in five persons did not have a CVD diagnosis. Each correct answer increases the cumulative proportion of the sample accounted for. A YES percentage of 39.8% accounted for 39.8% of the total sample. When 60.2% is included, the total percentage reaches 100.0%, reflecting the whole valid sample.

**Figure 1:**
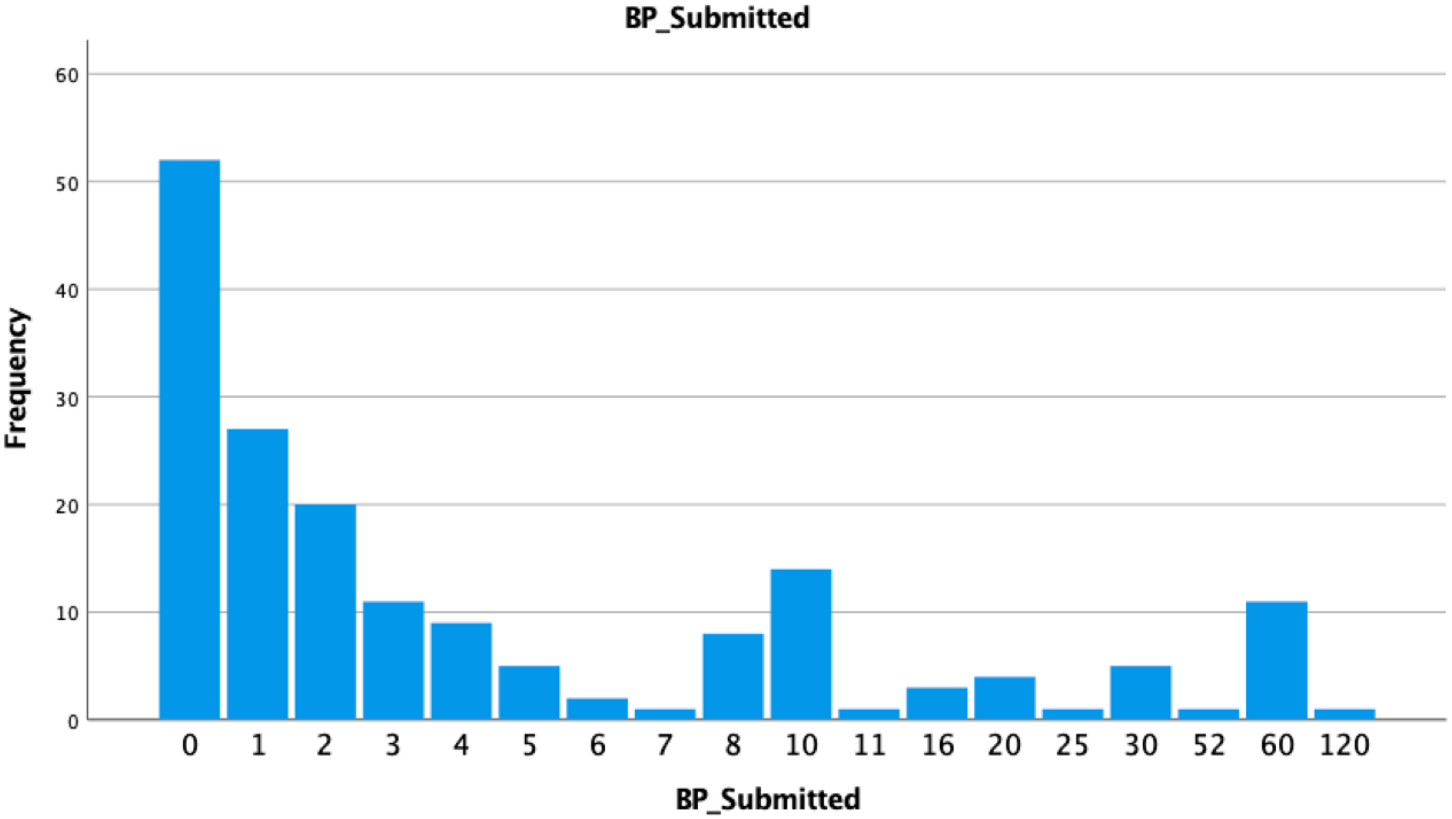
Frequency of blood pressure submission among non-app user.

The BP_Submitted data, comprising 176 individuals' blood pressure readings, ranged from 0 to 120, with higher values indicating more readings. Notably, all participants had home blood pressure monitoring systems. The most common response was 0 readings (52 participants, 29.5%), followed by one reading (27 participants, 15.3%) and two readings (20 participants, 11.4%). A majority (67.6%) submitted 0–4 readings. A few participants submitted a high number, with one submitting 120 readings. The data included 18 different values, reflecting varied engagement levels. The mean number of readings was 7.41, with a standard deviation of 17.35, indicating substantial variation. The frequency distribution of BP submissions among non-app users is provided in Table 7 and Figure 1.

**Table 7:** CVD frequency in non-app users.

CVD	Frequency	Percent	Valid percent	Cumulative percent
Yes	70	39.8	39.8	39.8
No	106	60.2	60.2	100.0
Total	176	100.0	100.0	

## 5 Discussion

The healthcare industry is undergoing transformation owing to the integration of digital health technology, which is replacing the antiquated “one-size-fits-all” approach to healthcare management with actual, precise, and individualized treatment (12, 13). The recent pilot research presented here offers early data supporting the feasibility and acceptability of a mobile health intervention for remote cardiac monitoring and management. Telemonitoring or remote cardiovascular monitoring can address at-risk patients with chronic conditions such as heart failure (14).

Our research sought to determine how well Romanian individuals with and without cardiovascular disease (CVD) utilized telemonitoring applications to measure their blood pressure over a two-month period. The average number of blood pressure submissions among the 24 individuals who used the telemonitoring app was 31.79, with a range of 2–60 submissions. A direct comparison of BP submission metrics between app users and non-users is shown in Table 8. The average number of blood pressure submissions among 176 patients who did not use the telemonitoring app and were solely reliant on the traditional method of blood pressure recording was 7.41, with a range of 0–120 submissions. The fact that there was such a large difference suggesting that patients who had access to the telemonitoring software were more dedicated to tracking and reporting their blood pressure data. The findings of our study show that telemonitoring applications might be beneficial in increasing compliance of blood pressure recordings in Romania. People who had access to the telemonitoring app used it much more than those who did not track and submit their blood pressure data. This is in line with our studies conducted before, it has been shown that individuals using mobile health (mHealth) apps for hypertension management are more likely to engage in frequent BP monitoring. For example, a study found conducted by Nakrys et al. showed that app users demonstrated better adherence to self-monitoring protocols, with active engagement leading to a higher likelihood of submitting regular BP readings and achieving better BP control outcomes (15, 16). In our study patients with telemonitoring app were consistently reminded to take their blood pressure readings from a blood pressure monitoring device and input into the telemonitoring app so that the data could be uploaded for the blood pressure. A constant reminder from the telemonitoring app was essential, as it enabled the patients to adhere to monitoring and input practices. These data confirmed our hypothesis that telemonitoring technology leads to better adherence to monitoring regimens in patients with confirmed CVD. A significant feature of digital health is the ability to provide personalized feedback, instructions, and encouragement using smartphone interfaces. Beyond merely counting step counts, the app could be expanded to include personalized physical activity programs and features such as reminders and prizes to assist in habit change. Similar customization might be integrated into the blood pressure monitoring components, using transmitted data to send timely warnings when high blood pressure is detected, along with lifestyle adjustment and medication adherence.

**Table 8:** BP submitted frequency.

BP_Submitted	Frequency	Percent	Valid percent	Cumulative percent
0	52	29.5	29.5	29.5
1	27	15.3	15.3	44.9
2	20	11.4	11.4	56.3
3	11	6.3	6.3	62.5
4	9	5.1	5.1	67.6
5	5	2.8	2.8	70.5
6	2	1.1	1.1	71.6
7	1	0.6	0.6	72.2
8	8	4.5	4.5	76.7
10	14	8.0	8.0	84.7
11	1	0.6	0.6	85.2
16	3	1.7	1.7	86.9
20	4	2.3	2.3	89.2
25	1	0.6	0.6	89.8
30	5	2.8	2.8	92.6
52	1	0.6	0.6	93.2
60	11	6.3	6.3	99.4
120	1	0.6	0.6	100.0
Total	176	100.0	100.0	

With regard to physical activity, the wristwatch offered objective monitoring of daily step count. On average, patients with CVD accumulated marginally more steps per day than those in the non-CVD group, 4,903 contrary to 4,385 steps respectively. However, both approaches fall short of the suggested minimum of 5,000 daily steps for health benefits. This study underlines public health concerns regarding widespread physical inactivity and highlights the need for lifestyle interventions to encourage more daily mobility (17). Although CVD may enhance risk awareness and motivation, professional help via digital coaching is likely essential for most patients to effectively alter ingrained sedentary behaviors.

Telemonitoring app users are more engaged, which suggests that this technology might greatly improve patient compliance and, therefore, cardiovascular outcomes. To prevent CVD effects, such as heart attacks and strokes, one must successfully monitor and control blood pressure. By providing users with a convenient tool to monitor their vital signs and share this information with healthcare professionals, telemonitoring apps can encourage early intervention and improve health outcomes. A systematic review and meta-analysis highlighted that app-based interventions not only improve BP monitoring frequency but also support lifestyle changes, such as dietary modifications and medication adherence by helping the individual stay active on the app and submit their daily readings, which contribute to better hypertension management (16).

These findings are in line with research from other nations that has shown the benefits of telemonitoring for cardiovascular health (18). Previous studies have shown a correlation between the use of telemonitoring in the context of CVD and improved blood pressure control, reduced hospital readmissions, and lower healthcare costs (19). However, from a systematic review by C Kruse, it has come to the knowledge that barriers to telemedicine vary greatly, and it is still not extensively used. The biggest obstacles are related to technology, but they might be eliminated with training, change-management strategies, and a combination of direct patient-to-provider contact and telemedicine (10). Furthermore, qualitative research conducted in Romania has indicated possible obstacles, which must be addressed, such as patient digital proficiency, privacy concerns, and preferences for in-person treatment (20).

## 6 Limitations

This research makes an important contribution by demonstrating greater blood pressure monitoring engagement among app users than among non-users. However, this study has some limitations. The sample size of 24 app patients and a 2-month follow-up period, while sufficient for an early study, limits the generalizability of the findings. As the duration of the current study was shorter, we aimed to increase the follow-up duration of the participants in the subsequent study. The comparison group of 176 patients who did not use the app also had limitations. This much larger sample size could have introduced bias when comparing the two groups, as this group was based on an online questionnaire. There is a lack of information on the benefits of apps from the perspective of participants, which we aim to explore in a subsequent study. Furthermore, having only grouped frequency data for their blood pressure submissions restricted the statistical comparisons with the intervention arm. Going forward, a matched sample size and individual-level data would provide more rigorous comparison. While our study provides initial evidence of improved adherence with telemonitoring, a comprehensive feasibility assessment including usability, user experience, and barriers remains essential for future implementation which shall be assessed in future research. The study may also involve a self-selection bias, as patients who volunteered for the app could be more motivated to self-monitor. Randomization of a larger sample could help control this. Finally, details on participants' specific cardiovascular diagnoses, demographics, medications, and comorbidities were lacking. Collecting such data would allow the analysis of how these factors influence app engagement and blood pressure outcomes.

## 7 Future implications

This study set the stage for expanded research on the utility of mobile health apps for blood pressure telemonitoring. Future directions include conducting randomized controlled trials over 6–12 months to compare the app and non-app groups more rigorously. Researchers should gather detailed data on patients' cardiac conditions, medications, comorbidities, and demographics to understand how these variables affect engagement and outcomes. Investigating usage patterns over longer periods can provide insights into the sustainability of app use for chronic disease management. Furthermore, other options to track blood pressure include wearable devices equipped with blood pressure monitoring capabilities, which offer several advantages: they provide patients with a convenient way to track their blood pressure regularly, allowing for more proactive management. Additionally, wearable blood pressure devices or wearables with built-in blood pressure functionality can synchronize data with smartphones or other devices, making it easier for patients to share their information with healthcare providers and thus promoting better adherence to treatment plans. In the future, we would like to further explore the specific benefits and challenges of wearable blood pressure monitoring and its role in increasing patient engagement in subsequent studies. We aimed to explore the reasons for the decreased input of blood pressure readings in patients with the app. In addition, the lack of evidence-based digital health standards, privacy, data security, and confidentiality concerns prevent the implementation of this service (21).

Surveying users and non-users could shed light on perceived advantages, disadvantages, and barriers to adoption of the technology. Ethical challenges such as the need to ensure that digital health technologies are used in a way that respects patients' autonomy, privacy, and confidentiality are essential for widespread adoption, and this must be looked into (22).

Cost-effectiveness analyses also demonstrated the value of telemonitoring apps for improving health while reducing costs. Beyond patients with known cardiovascular disease, expanding research to those with hypertension but no diagnosed CVD could evaluate the prevention potential of self-monitoring apps. Testing the integration of the app with electronic health records and clinician workflows could facilitate its broader implementation. Addressing these areas will provide the necessary evidence for mobile health apps as an effective approach to managing heart health through patient engagement in their own care.

Furthermore, as digital health evolves, advanced data science tools can extract greater value from patient-generated data. In this work, the authors suggest employing artifithe cial intelligence algorithms to forecast the risk of the heart failure exacerbations based on the gathered measures. Machine learning algorithms can discover complicated patterns and early warning flags that are unnoticeable to human analysis. Enabling predictive analytics might enable prompt therapeutic intervention and the prevention of catastrophic deterioration. Although massive data gathering over a long period may be necessary to train effective algorithms, this demonstrates the future promise of digital health advancements.

## 8 Conclusion

This pilot study suggests that mobile health technology may enhance patient engagement in hypertension management in Romania, as evidenced by significantly higher blood pressure submission rates among app users compared to non-users. While these preliminary findings are encouraging, the small sample size and heterogeneity of participants necessitate cautious interpretation. The results align with emerging evidence on telemonitoring's potential to support cardiovascular health, though further research with larger, more representative cohorts is needed to validate these observations. This study contributes to the growing body of literature on digital health solutions by demonstrating improved adherence to self-monitoring which is a critical first step in hypertension management. However, the clinical impact on cardiovascular outcomes remains to be established.

## Data Availability

The original contributions presented in the study are included in the article/Supplementary Material, further inquiries can be directed to the corresponding author.
